# An ethnobotanical survey of medicinal plants used in Terai forest of western Nepal

**DOI:** 10.1186/1746-4269-8-19

**Published:** 2012-05-16

**Authors:** Anant Gopal Singh, Akhilesh Kumar, Divya Darshan Tewari

**Affiliations:** 1 Department of Botany, Butwal Multiple Campus, Tribhuvan University, Tribhuvan, Nepal; 2 Eco-Auditing Group, CSIR-National Botanical Research Institute, Rana Pratap Marg, Lucknow, 226001, India; 3 Department of Botany, Maharani Lal Kunwari Post Graduate College, Balrampur, Uttar Pradesh, India

**Keywords:** Ethnobotany, Medicinal plants, Traditional healers, *Tharu*, *Magar*, Terai forest, Nepal

## Abstract

**Background:**

Nepal Himalayas have been known as a rich source for valuable medicinal plants since Vedic periods. Present work is the documentation of indigenous knowledge on plant utilization as natural remedy by the inhabitants of terai forest in Western Nepal.

**Methods:**

Study was conducted during 2010–2011 following standard ethnobotanical methods. Data about medicinal uses of plants were collected by questionnaire, personal interview and group discussion with pre identified informants. Voucher specimens were collected with the help of informants, processed into herbarium following standard methods, identified with the help of pertinent floras and taxonomic experts, and submitted in Department of Botany, Butwal Multiple Campus, Tribhuvan University, Nepal for future references.

**Results:**

During the present study 66 medicinal plant species belonging to 37 families and 60 genera has been documented. These plants were used to treat various diseases and ailments grouped under 11 disease categories, with the highest number of species (41) being used for gastro-intestinal disorders, followed by dermatological disorders (34). In the study area the informants’ consensus about usages of medicinal plants ranges from 0.93 to 0.97 with an average value of 0.94. Herbs (53%) were the primary source of medicine, followed by trees (23%). *Curcuma longa* (84%) and *Azadirachta indica* (76%) are the most frequently and popularly used medicinal plant species in the study area. *Acacia catechu, Bacopa monnieri, Bombax ceiba, Drymaria diandra, Rauvolfia serpentina*, and *Tribulus terrestris* are threatened species which needs to be conserved for future use.

**Conclusions:**

The high degree of consensus among the informants suggests that current use and knowledge are still strong, and thus the preservation of today's knowledge shows good foresight in acting before much has been lost. The connections between plant use and conservation are also important ones, especially as the authors note that neither the local inhabitants nor the government is addressing the potential loss of valuable species in this region.

## Background

The *Rig-Veda* written during 4500 BC to 1600 BC is believed to be the oldest repository of human knowledge about medicinal usages of plants in Indian subcontinent. In Nepal, although such old documentation is still not rediscovered, but the knowledge on plant utilization is believed to be very old. According to WHO [1], about 80% of the world’s population, especially in the rural areas depends on herbal medicine for their healthcare needs. About 90% of the Nepalese people reside in rural areas where access to government health care facilities is lacking [2]. The ethnic people residing in different geographical belts of Nepal depends on wild plants to meet their basic requirements and all the ethnic communities have their own pool of secret ethnomedicinal and ethnopharmacological knowledge about the plants available in their surroundings [2-20], which has been serving rural people with its superiority. Due to changing life style, extreme secrecy of traditional healers and negligence of youngsters, the practice and dependence of ethnic societies in folk medicines is in rapid decline globally, therefore, ethnobotanical exploitation and documentation of indigenous knowledge about the usefulness of such a vast pool of genetic resources is deliberately needed [21-30]. We selected Terai forest of Rupandehi district and adjoining areas for ethnomedicinal investigation because this area is very rich in phytodiversity and tribal population. Besides other usages of plants the practice of oral tradition for healthcare management of human and domesticated animals using herbal medicines is still prevalent among the inhabitants of the area. They have enormous knowledge about medicinal uses of plants and this knowledge is mostly undocumented and transmitted orally from generation to generation. Recently due to unplanned developmental programs, increasing modern healthcare facilities and impact of modern civilization in this area, natural resources as well as traditional knowledge and tribal cultures are depleting rapidly at an alarming rate. Therefore, it is urgent to explore and document this unique and indigenous, traditional knowledge of the tribal community, before it diminishes with the knowledgeable persons. Further, documentation of indigenous and traditional knowledge is very important for future critical studies leading to sustainable utilization of natural resource and to face the challenges of bio-piracy and patenting indigenous and traditional knowledge by others. Besides, to the best of our knowledge no ethnobotanical work has been carried out in this area. Keeping these things in mind present study was proposed to document the ethnomedicinal knowledge in terai forest of western Nepal. Aims of the present study are:

(A) Identification and documentation of plant species used for the treatment and prevention of various diseases and ailments in the study area.

(B) Identification of most common and popularly used medicinal plant species for the treatment and prevention of various diseases and ailments in the study area.

(C) Find out the level of consensus agreement between the informants regarding the uses of particular medicinal plant(s) for the treatment of particular disease category.

### Study area

**Figure 1:**
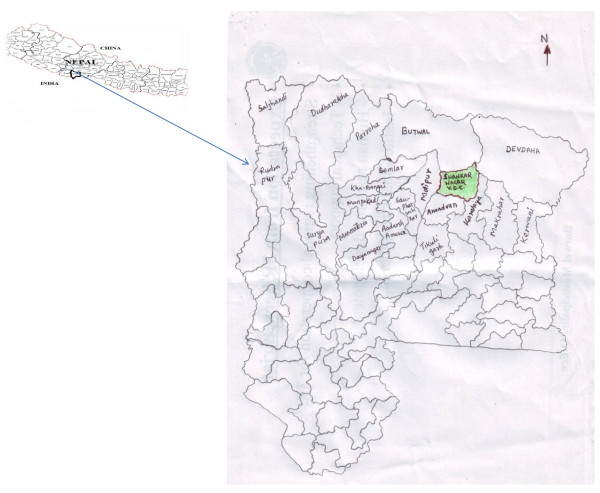
Location map of study site.

Rupandehi district is situated in the Terai region of western Nepal. It lies between 83^0^27'.955" to 83^0^28'.255" E and 27^0^40'.016" to 27^0^40'.252" N geographical limits in 1360 Km^2^ area at altitudinal variation from 105 to 258 meters. Rupandehi district (Figure 1) is surrounded by hilly districts (Palpa and Arghakhanchi) in North, by Mahrajganj district of Uttar Pradesh (India) in south, by Nawalparasi district in East and by Kapilvastu district in west. It has tropical climate with maximum temperature beyond 40^0^C during summer (May- June) and below 10^0^C during winter (December- January) and annual rainfall is about 1250 mm. Geographically, it is divided into Chure region (14.5%); Bhabar region (0.6%) and Terai region (84.9%). The famous river and rivulets of this district are Tinau, Rohini, Danaw, Pahela, Kanchan, Kothi, Danda, Koili etc. All the rivers flow from north to south. The climatic condition of the study site is tropical type and predominated by Sal forest. The forest area of the district is divided into community forest, religious forest and personal forest [31]. The vegetation of the study is dominated by sal (*Shorea robusta*) forest along with sissoo (*Dalbergia sissoo*), saj (*Terminalia alata*) khayar (*Acacia catechu*), baheda (*Terminalia bellirica*), dabdabe (*Garuga pinnata*), khaniyu (*Ficus semicordata*), asuro (*Justica adhatoda*), dhaiyaro (*Woodfordia fruticosa*), and titepati (*Artemesia indica*) etc. The main highway Siddhartha Rajmarga runs from the middle part of Shankar Nagar VDC. All the parts of Shankar Nagar VDC and its surrounding areas are interconnected by network of road and are easily accessible for the field visits.

### Ethnography

The *Tharu* and the *Magar* are the main ethnic societies of the study area. They live in association with Chhetri, Brahmin, Thakuri, Gurung, Damai, Kumal, Bote, Majhi, Mushahar, Kami, Newar and others communities. Total population of the district was 7, 08,419 [32] The *Tharu* tribal community share 10.57% population of the district [31]. They are scattered all along the southern foot hills of the Himalayas. The greater parts of their population resides in Nepal, although they are also scattered in the adjacent Indian district of Champaran, Maharajganj, Gorakhpur, Siddharthnagar, Basti, Balrampur, Baharaich, Shravasti, Lakhimpur-Kheri, and Nainital. There are several endogamous sub groups in the Tharu community, such as Rana, Kathuria, Dangauria, Kochila, and Mech. Tharu people choose plain lands at the jungle side or river side for house construction. They like to settle in the group of their own community members, thus their houses are found dense within a small area. *Tharu* people used to live in joint family traditionally and it is practiced up to now. In Tharu village, the duty of maintaining good relations among villagers, as well as conducting the village’s affairs, falls on the Mahaton (Village chief). A mahaton is elected by Gardhurryas (Tharu house hold chief) from among themselves. A Mahaton is elected, but once elected; the office becomes hereditary, unless a particular incumbent is considered a misfit. The assembly of Gardhurryas can remove an unsuccessful Mahaton. The role of mahaton in the assembly of Gardhurryas is like that of a chairman and a judge who keep others view in mind, gives the final communal decision. Due to their own believes, judgement policy and living together in close vicinity, they are considered as native Tribal community of Terai region. In Nepal *Tharu* tribal community is settled in the southern part of the country from the east to west along Indo-Nepal boarder and the adjacent valleys and plains between the Chure hilly regions. The *Tharus* are famous for their ability to survive in the moist Terai region which is deadly to outsiders due to malaria. They are farmer by occupation and cultivate rice, mustard, corn and lentils but also collect forest products such as wild fruits, vegetables, medicinal plants and material to build their houses, hunt wild animals and fishes [33].

## Materials and methods

### Field works and collection of data

Field studies were conducted from March 2010 to May 2011. Methods of Martin [34] were followed for the collection of data and voucher specimen during the field study. First of all local administrative officers were consulted with the explanation of aims and objectives of the research for the identification of resource persons (informants). They give advice regarding the people who would be the best sources of information. Researchers meat these peoples and explain the research theme. These informants often suggested other potential informants. In order to insure a sample that includes representatives of whole community, we attempted to interview peoples from variety of age groups, sex, socio-economic and ethnic community (for detail information about gender, age, ethnicity, and occupation of informants please see Table 1). The criteria for the selection of informants for the interview were their reputation in the society regarding their knowledge about herbal medicines and traditional healthcare system. Total 55 informants were identified from Shankar Nagar VDC and surrounding areas. They are reputed knowledgeable persons of the society and the collected data from these informants represent the whole community, because they are knowledgeable healers, villagers, senior citizens, teachers, social workers etc. Prior to survey, a questionnaire was designed and pre-tested with five informants to find out its suitability for present study and modified according to response of informants. The revised questionnaire was used for gathering data about medicinal plants of the study area. Pre informed consent was obtained from the resource persons before interview. Field survey was conducted taking traditional healers as a guide and voucher specimens of cited medicinal plants were collected and their local identity was re-confirmed by other informants. During data collection three visits (in each visit author stay for four days in study area) was conducted and information’s were collected. The information obtained was cross checked with the other informants. The local names, habit, wild/cultivated, availability of medicinal plants, need of conservation and efforts made by inhabitants and traditional medicinal uses of plants were carefully recorded. Finally, group discussion ware made with the healers and local people to know their perception about the use of traditional folk medicines, awareness about the conservation of phytodiversity and indigenous knowledge.

**Table 1:** Detail of informants interviewed in terai forest of western Nepal

**S N**	**Name**	**Age (Y)**	**Sex**	**Address**	**Occupation**
1	Durga Pd Shrestha	69	M	Butwal-12 Kalikanagar, Rupandehi	Senior citizen, knowledgeable person
2	Mohan Lal Tharu	26	M	Motipur-5, Rupandehi	Plant collector
3	Babu Ram Rana	65	M	Paschim Amuwa-5, Rupandehi	Local healer, farmer
4	Laxman Aryal	43	M	Shankar Nagar-4, Rupandehi	Secretary, VDC, Shankar Nagar
5	Khadanand Poudyal	58	M	Shankar Nagar-4, Rupandehi.	Shopkeeper
6	Bhagirathi Tharu	63	M	Motipur-5, Rupandehi	Local healer (vaidya), farmer
7	Indra Bdr. Bhujel	39	M	Paschim Amuwa-4, Rupandehi	Fodder collector, farmer
8	Krishna Bdr. Rana	64	M	Ram Nagar Butwal-12, Rupandehi	Plant collector, farmer
9	Harka Bdr. Rasaily	47	M	Semlar-7, Rupandehi	Local healer, farmer
10	Yam Bdr. K. C.	52	M	Manpakadi-8, Rupandehi	Fodder expert, healer, farmer
11	Indrawati Tharuni	58	F	Sou. Pharsatikat-4, Rupandehi	Local healer, farmer
12	Khadak Thapa	44	M	Shankar Nagar-9, Rupandehi	Plant collector, farmer
13	Kishuni Tharuni	49	F	Dudhraksh- 5, Rupandehi	Plant collector, local healer
14	Yam Bdr Thapa Magar	54	M	Saljhandi-4, Rupandehi	Local healer, plant collector
15	Top Naarayan Ghimire	56	M	Motipur-4, Rupandehi	Secretary of chartapa irrigation, local healer, farmer
16	Ram Kumari Chai	58	F	Sikthan-4, Rupandehi	Active women farmer
17	Sher Bdr. Budhathoki	73	M	Kalika Nagar, Butwal-12, Rupandehi	Senior citizen, farmer
18	Gopal Pd. Neupane	69	M	Shankar Nagar-1 Chaparhatti, Rupandehi	Local knowledgeable healer, farmer
19	Khushi Lal Tharu	58	M	Motipur-5, Rupandehi	Plant collector, local healer (vaidya)
20	Narjeet Tharu	53	M	Motipur-7 Rupandehi	Local healer, farmer
21	Rajendra Lodh	47	M	Shankar Nagar-3, Rupandehi	Local healer, farmer
22	Nar Bdr. G. M.	52	M	Gopalpur, Kha Bangai-4 Rupandehi	Farmer, local healer
23	Sun Bdr. Gaha	67	M	Koldanda-1 Lagad, Palpa	Local Healer, head of Lagad Village, Palpa.
24	Luk Bdr. Gaha	58	M	Koldanda-1 Lagad, Palpa	Plant collector and exporter.
25	Punam Kunwar	33	F	Butwal-12, Rupandehi	Secretary, Butwal −12, service.
26	Laxmi Narayan Chaudhary	48	M	Parroha-2 Rupandehi	Farmer, local healer
27	Nar Bdr. Rana	49	M	Shital Nagar, Devdaha, Rupandehi	School teacher, farmer
28	Ram Ratan Gupta	58	M	Siloutiya 5 Marchwar, Rupandehi	Head master, secondary school, odwalia
29	Ganga Kharel	47	F	Shankar Nagar-3 Rupandehi	Health assistant
30	Bhim Pd Neupane	66	M	Motipur- 4 Rupandehi	Senior citizen, Ex VDC Chairman
31	Yam Bdr G.M.	43	M	Makrahar-6 Rupandehi	Local healer
32	Hare Ram Yadav	55	M	Motipur-2, Rupandehi	Mukhiya, Panchmauja, Chartapa irrigation, farmer
33	Mrs. Janaki Aryal	48	F	Motipur-7, Rupandehi	Social worker, farmer
34	Salik Ram Aryal	64	M	Sou-pharsatikar-1, Rupandehi	Senior citizen, Ex VDC chairman
35	Mrs. Rita Wasti	52	F	Motipur-7, Rupandehi	Farmer and knowledgeable woman
36	Krishna Chand Chaudhary	59	M	Kha-Bangai-4, Rupandehi	Local healer, farmer
37	Durga Khanal	58	M	Semlar-3, Rupandehi	Secretary of Semlar VDC
38	Chhabi Lal Neupane	64	M	Motipur-7, Rupandehi	Mukhiya, farmer
39	Chautare Pd. Tharu	72	M	Motipur-7, Rupandehi	Local healer,farmer, member of 11 mauja irrigation, chartapa
40	Krishna Kumar Thapa	38	M	Motipur-2, Rupandehi	Plant collector
41	Om Prakash Aryal	38	M	Motipur-9, Rupandehi	Farmer, plant collector
42	Ishwar Raj Lamsal	58	M	Butwal −10, Deepnagar, Rupandehi	Knowledgeable person
43	Om Prakash Chaudhary	52	M	Butwal −13, Devinagar, Rupandehi.	Knowledgeable local healer & Farmer
44	Sohan Lal Chaudhary	48	M	Karhiya-2, Rupandehi	Plant collector & Farmer
45	Ram Prasad Tiwari	62	M	Tikuligadh-3, Rupandehi	Local Plant collector
46	Krishna Mohan Kohar	42	M	Basantpur-2 Rupandehi	Knowledgeable person, teacher
47	Rammu Prasad Chaudhary	65	M	Piparanhwa, Baguali-3, Rupandehi	Knowledgeable person
48	Kamal Narayan Chaudhary	55	M	Anandban VDC-4, Rupandehi	Knowledgeable person
49	Bhiku Prasad Chaudhary	46	M	Kalika Nagar, Butwal-12, Rupandehi	Knowledgeable person
50	Dil Bahadur Mukhiya	42	M	Kalika Nagar Butwal- 13, Rupandehi	Plant collector
51	Mandali Tharu	56	M	Kha- Bangai-6, Rupandehi.	Local healer, Gurau
52	Tameshwar Tharu	52	M	Gopalpur, Kha- Bangai-2, Rupandehi	Local healer, plant collector
53	Harihar Tharu	48	M	Bhujauli, Kha- Bangai-7, Rupandehi	Local healer, plant collector
54	Tulshi Prasad Chaudhary	53	M	Kha-Bangai-6, Rupandehi	Knowledgeable person
55	Khadag Bahadur Mahat	51	M	Kalika Nagar Butwal-12, Rupandehi	Knowledgeable person, social worker

S N = serial number; Y = years; M = male; F = female.

### Processing of voucher specimens for herbarium preparation and identification

The voucher specimens were brought to the laboratory and processed for herbarium specimen preparation [34-36] and identified with the help of available floras and other pertinent literatures [8,11,23,37-42] and submitted in department of Botany, Butwal Multiple Campus, Tribhuvan University, Nepal for future references. The botanical identities of collected specimens were confirmed by Dr. M. P. Panthi, and Mr. B. R. Nepali, Taxonomist, Tribhuvan University, Kathmandu, Nepal. Plant names were checked according to International Plant Name Index [43].

### Statistical analysis

The data were spreads on Excel sheet to summaries and to identify various proportions like plant families, habit, availability of medicinal plants, plant parts used as medicine, methods of use, frequency of citation and popularly used medicinal plants in the study area. Frequency of citation was calculated by following formula-

(1) $$\text{Frequency of citation(\%)}=\frac{\text{Number of informats who cited the species}}{\text{Total number of informats interviewed}}\times 100$$

Factor of informants consensus (F_IC_) for different ailment categories was calculated for testing homogeneity on the informant's knowledge followed by the method provided by Trotter and Logan and Heinrich et al. as under [44,45].

(2) $${\text{F}}_{\text{IC=}}\frac{{\text{N}}_{\text{UR}}-{\text{N}}_{\text{TAXA}}}{\left({\text{N}}_{\text{UR}}-1\right)}$$

Where N_UR =_ number of use report in a particular illness category and N_TAXA =_ number of taxa used to treat that particular category by informants.

## Result and discussion

### Medicinal plants and their uses

Altogether 66 medicinal plants belonging to 37 families and 60 genera were documented from the study area (Table 2). The documented medicinal plants and their ethnomedicinal uses along with common name have been summarized in Table 2. These plant species are used for the treatment and prevention of many ailments and diseases grouped under 11 ailment categories (Table 3). The common sickness for the tribal in the study area are cold, cough, bronchitis, diarrhoea, dysentery, gastritis, headache, backache, cuts, wounds etc. Symptoms of the diseases given by the tribes in local language with their bio-medical terms are given in Table 4. Exact doses and duration of treatment are considered as intellectual property of informants, so as per their request this information is not included in the present paper. *Curcuma longa* (84%), *Azadirachta indica* (76%) are the most frequently and popularly used medicinal plant species in the study area.

**Table 2:** Ethnomedicinal plants of Terai forest in western Nepal and their traditional therapeutic uses

**Botanical name, family, voucher no.**	**F**	**Local name/habit/availability**	**Parts used**	**Usages**
*Acacia catechu* (L.f.) Willd., Fabaceae, AGS-45	22	Khayar (N/M)/Tree/Wild/Rare	Bark, wood	*Bark powder is applied on tooth ache. *Wood decoction is given orally in intestinal pain. Bark paste is applied in skin diseases.
*Acalypha indica* L., Euphorbiaceae, AGS-66	15	Mukta barshi jhar (N),/Herb/Wild/Easily	Entire plant	*Plant decoction is given orally in toothache and earache. *Leaf paste is applied on burns. *Fresh leaf juice is applied on rheumatoid arthritis.
*Acorus calamus* L. Acoraceae, AGS-71	27	Bojho (N)/Katara (Th)/Herb/Wild	Root	Juice of root is given orally in stomach disorders, bronchitis, fever and its small piece chewed to clear the throat and open the voice.
*Achyranthes aspera* L. Amaranthaceae, AGS- 33	33	Ulta chirchiri (Th)/Datiwan (N)/Herb/Wild/Easily	Entire plant	Decoction of plant is given as diuretic. Root juice is applied to treat toothache. *Root juice is given orally asthma. Stem is used as toothbrush in tooth problems.
*Aegle marmelos* (L.) Correa ex Roxb., Rutaceae, AGS- 25	20	Bel (N/Th) /Tree/Cultivated	Fruit pulp, leaf and root	Fruit juice is given orally in Diarrhoea and dysentery. Leaves are given orally in stomach disorders. *Root juice is given orally in fever and vomiting.
*Ageratum conyzoides* L. Asteraceae, AGS-49	38	Gandhe jhar (N)/Gandhaula (Th)/Herb/Wild/Easily	Leaf	Leaf juice is given to cure bleeding from cuts and wounds. Plant paste is applied to cure muddy wounds between toes during rainy season.
*Aloe vera* (L.) Burm.f. Aloaceae, AGS- 38	35	Ghiu Kumari (N)/Ghrit Kumari (Th) Herb/Cultivated	Leaf juice Leaf pulp	*Leaf pulp is given orally in lung disease and stomach disorders. Leaf pulp is applied on skin burns.
*Amaranthus spinosus* L. Amaranthaceae, AGS-15	38	Ban lunde (N)/Kande Lundo (M)/Herb/Wild, easily	Tender shoot, root	Root decoction is given as diuretic. Tender shoot is given to cure leucorrhoea, flatulence, and colic pain.
*Argemone mexicana* L. Papaveraceae, AGS-11	36	Bharbhanda (Th)/Herb/Wild, easily	Milky juice and root	Milky juice of the plant is applied on tumors and skin diseases. Root paste is applied on skin diseases and flatulence.
*Artemisia indica* Willd. Asteraceae, AGS-52	18	Tite pati (N)/Pati (Th)/Herb/wild or cultivated	Tender shoot and leaves	*Leaf juice is given orally in bronchitis. Leaf paste is applied in skin diseases. Dried tender shoot powder is given orally in fever.
*Asparagus racemosus* Willd*.,* Liliaceae, AGS-28	31	Kurilo(N)/Santawar (Th)/Herb/cultivated	Tuberous root	Dried root powder is given orally with hot water to cure urinary troubles. Root decoction is given orally after delivery as tonic. Tuberous root powder is given orally to increase lactation.
*Azadirachta indica* A. Juss., Meliaceae, AGS- 8	76	Neem (N/M/Th)/Tree/Wild and cultivated	Tender shoot, leaf and bark/Tree	Decoction of fresh leaves is used to wash skin to treat scabies. Young stem is used as tooth brush in tooth problems. Fresh leaves are given orally for the purification of blood and for control of sugar level. Tender twigs paste is applied on wounds for early healing.
*Bacopa monnieri* (L.) Pennel, Scorphulariaceae, AGS-21	16	Jal nim, Brahami(N)/Khole Sag (M/Th)/Herb/Wild/Easily in northern parts, rare in southern parts	Entire plants/Herb	Plant juice is given orally as diuretic, cardiac tonic and memory enhancer. Plant juice is given as hair tonic especially in thinning and falling hairs.
*Bauhinia variegata* L. Fabaceae, AGS-68	42	Koiralo (N)/Koilar (Th)/Tree/cultivated	Bark and flower juice	*Bark decoction and flower juice are given in Diarrhoea, dysentery, indigestion and body ache. *Bark decoction is given to cure tumors.
*Bombax ceiba* L. Bombacaceae, AGS- 35	22	Simal (N)/Semar (Th)/Tree/Wild/Rare	Root	Root decoction is given as tonic, anti-dysenteric and in urinary troubles. *Bark decoction is given orally in bronchial diseases.
*Calotropis gigantea* (L.) W.T. Aiton, Asclepiadaceae, AGS-12	15	Aank (N)/Madar (Th)/Shrub/Wild/Easily	Root, Milky latex and flower/Shrub	Root paste applied on boils, pimples, and skin disease. Milky latex is applied on muscular pain, cut, wounds, boils, and ringworm. *Flower powder is given orally in cough, cold, and bronchitis.
*Carica papaya* L. Caricaceae	18	Mewa (N)/Papita, Larmewa (Th)/Shrub/cultivated	Latex and fruit	*Milky latex is given in toothache and dysentery.
*Centella asiatica* (L.) Urb. Apiaceae, AGS- 36	29	Ghod Tapre (N)/Ghortapya, Bhatbhate(Th)/Herb/wild	Entire plant	Plant decoction is given orally as diuretic, tonic, blood purifier and in skin diseases, leprosy, and mental disorder. Leaf juice is given orally in indigestion.
*Chenopodium album* L. Chenopodiaceae, AGS-9	27	Bethe (N)/Bethuwa (Th)/Herb/Wild/Easily	Tender shoot and flower	Tender shoot and flower juice is given orally to kill and expel the round worm and in constipation.
*Citrus limon* (L.) Burm. f. Rutaceae,	49	Kagati (N)/Nibuwa (Th)/Shrub/Cultivated	Leaves and fruit	*Leaves are chewed to expel intestinal worms. *Rind paste and fruit juice is applied in pimples and dandruff.
*Colocasia esculenta* (L.) Schott, Araceae, AGS-19	20	Pindalu, Karkalo (N)/Gabha, Ghuiya (Th)/Herb/cultivated	Corm and tender aerial parts	*Petioles used as green vegetables in liver problems. *Corm paste is applied over cuts/wounds to stop bleeding.
*Coriandrum sativum* L. Apiaceae	18	Dhaniya (N/M/Th)/Herb/cultivated	Leaf and seeds	*Leaf paste is applied on allergic inflammation. *Green leaves are used in the preparation of soft drink along with sugar and given orally inx stomachache.
*Curcuma longa* L., Zingiberaceae	84	Besar (N)/Hardi (Th)/Herb/Cultivated	Rhizome	Rhizome decoction is given as stimulant, tonic, and blood purifier. Rhizome paste is externally applied on strains, wounds, and injuries. Fresh rhizome juice is given as anthelmintic. Rhizome powder is given orally with luck warm water in jaundice and liver disorders.
*Curcuma amada* Roxb. Zingiberaceae, AGS-34	31	Aama haldi (N)/Amaadi (Th) /Herb/ Cultivated	Rhizome/Herb	*Rhizome powder is given as digestive to clean throat and tongue. *Rhizome paste is externally applied on strains, rheumatism, and inflammation.
*Cuscuta reflexa* Roxb. Convolvulaceae, AGS- 65	53	Aakashbeli (N)/Baora (Th)/Parasitic climber/wild	Entire plant	*Juice of the plant is given orally to treat fever. Plant paste is applied externally to treat headache, stomachache and rheumatism. Plant paste is applied on fractures.
*Cymbopogon citratis* (DC. ex. Nees) Stapf, Poaceae, AGS-50	16	Pire ghans (N)/Kagati ghans(Th)/Herb/Cultivated	Leaves	Leaves are used to make tea and given orally in cough, cold, headache, and fever.
*Cyperus rotundus* L. Cyperaceae, AGS-48	20	Mothe (N)/Bhada (Th)/Herb/Wild/Easily	Tuber	Tuber infusion, with sugar/salt is given orally in dysentery, Diarrhoea, and indigestion and as anti inflammatory agent.
*Cynodon dactylon* (L.) Pers. Poaceae, AGS-5	27	Dubo (N)/Dub (Th)/Herb/Wild/Easily	Entire plant	Plant paste is applied on cuts and wounds. *Root infusion along with sugar is given orally in bleeding piles and indigestion. *Plant juice used as eardrop in earache.
*Dalbergia sissoo* Roxb., Fabaceae, AGS-40	18	Sisau (N)/Sisava (Th)/Tree/cultivated and wild	Bark and leaf juice	Bark and leaf juice are given orally in Diarrhoea, dysentery and as anthelmintic. It is applied externally on cut and wounds. *Leaf decoction is given orally in gonorrhea.
*Datura metel* L. Solanaceae, AGS-3	16	Kalo Dhaturo (N/M)/herb/Wild/Easily	Leaf, stem and seed	Leaves juice is given orally in epilepsy. *Dried stem and leaves are smoked in asthma. *Seeds are boiled in mustard oil and massaged on joint pains.
*Dendrocalamus hamiltonii* Nees & Arn. ex Munro, Poaceae, AGS-67	27	Tama Bans (N/M)/Shrub/Wild/Easily	Tender shoot and stem node	*Stem node paste is applied on boils. *Tender shoots (Tama) is consumed as vegetable as aphrodisiac.
*Dioscorea pentaphylla* L., Dioscoreaceae, AGS-32	18	Bhyakur (N)/Ban Tarul (Th)/Climber/Wild	Rhizome	*Boiled Rhizome is given orally in abdominal pain.
*Drymaria diandra* Blume Caryophyllaceae, AGS-64	16	Abijalo (N), Sirbire Jhar (Th)/Herb/Wild/Rare	Entire plant	Root juice is inhaled to treat sinusitis.
*Eclipta prostrata* L*.* Asteraceae, AGS-6	22	Bhringi jhar, Bhringraaj (N)/Bhangaraila (Th)/Herb/Wild/Easily	Entire plant	Plant paste is applied on cut, wounds, skin diseases, and pimples.
*Euphorbia hirta* L*.* Euphorbiaceae, AGS-22	33	Dudhe Jhar (N)//Doodhe Jharra (Th) Herb/Wild/Easily	Entire plant	Plant juice is applied on cuts and wounds. Leaf juice is given orally in diarrhoea.
*Ficus benghalensis* L., Moraceae, AGS-30	29	Bar (N)/Bargad (Th)/Tree/Wild/Easily	Bark and milky latex/Tree	Bark infusion is given orally in diabetes. *Milky latex is applied on muscular pain.
*Gloriosa superba* L., Liliaceae, AGS-31	38	Karihari, Kewari (N)/Climber/Wild/Easy		*Rhizome paste is applied externally on ringworm and other skin diseases.
*Ipomoea aquatica* Forssk., Convolvulaceae, AGS-16	40	Kerunga Sag (N)/Kermua Sag, Karmaiya Sag (Th)/Herb/Wild/Easily	Tender shoot	*Tender shoot is used as vegetable in gastric trouble and general debility.
*Ipomoea batatas* (L.) Lam., Convolvulaceae	16	Shakarkand (N)/Herb/Cultivated	Tuberous root and leaf juice	*Leaf juice is given orally in diabetes.
*Ipomoea carnea Jacq. ssp. fistulosa (Mart. ex Choisy) D. Austin*, Convolvulaceae, AGS-23	16	Behaya (Th),Besharam (N)/Shrub/Wild/Easily	Latex of leaf and tender shoot	*Latex of leaf and tender shoot are applied as antiseptic on wounds between toes in rainy seasons.
*Justicia adhatoda* L., Acanthaceae, AGS-14	42	Asuro (N)/Ross (Th)/Shrub/Wild/Easily	Leaf	Warm decoction of the leaves is given to treat asthma. Juice of fresh leaves along with honey is given orally as expectorant. Juice of leaf is inhaled in bleeding nose (sinusitis). Dried powder of entire plant parts is given in bronchitis and cough.
*Lagenaria siceraria* (Molina) Standl., Cucurbitaceae	18	Lauka (N)/Climber/Cultivated	Leaf, fruit and seed	*Leaf decoction with sugar is given in jaundice. Fruit juice is given in diarrhoea and, dysentery *Seeds are given as mental tonic.
*Lepidium sativum* L., Brassicaceae, AGS-63,	33	Chamsur (N/Th)/Herb/Cultivated and wild	Entire plant	Seed paste is applied on rheumatism. *Fresh leaves are given orally in liver problems.
*Linum usitatissimum* L., Linaceae,	18	Tishi (Th), Alasa (N)/Herb/Cultivated	Seed, and seed oil	Seed oil is applied on burns and boils. *Seed poultice is applied on rheumatic and swellings.
*Malva parviflora* L., Malvaceae, AGS-31	25	Laphe sag (N)/Bariyara (Th)/Herb/Wild/Easily	Tender shoots & Seeds	Decoction of seeds is given orally in cough and bronchitis. Tender shoots are given orally to treat swollen glands of throat. *Decoction of tender shoot and seeds are given orally to control irregular menstrual cycle.
*Melia azadirachta* L., Meliaceae, AGS- 41	60	Bakaino (N)/Bakain (Th)/Tree/Wild/Easy	Entire plant	Root decoction is given orally as blood purifier. Leaf paste is applied on scabies. Poultice of flower is applied externally in skin eruption.
*Mentha spicata* L., Lamiaceae, AGS-18	38	Pudina (N)/Patina (Th)/Herb/Cultivated	Entire plant	Leaves decoction is given orally to cure throat infection and indigestion. *Decoction of leaves with cinnamon is given orally to women for easy delivery.
*Mimosa pudica* L., Fabaceae, AGS-60	20	Boohari Jhar (N)/Lajjalu Jhar (Th)/herb/Wild/Easily	Entire plant	*Leaf paste is applied on hydrocele. *Leaf and root paste are given orally in piles. Decoction of plant is given in Diarrhoea, dysentery and leucorrhoea.
*Mucuna pruriens* (L.) DC., Fabaceae, AGS-53	27	Kauso (N)/Kewanch (Th) Climber/Wild/Easily	Roots, fruits and seeds	Root decoction is useful in frequent urination. *Leaf decoction is given orally in weakness and headache.
*Musa paradisiaca* L., Musaceae	18	Kera (N/M/Th)/Shrub/Cultivated	Leaf, flower and fruit	Unripe fruits are roasted and given orally in Diarrhoea and dysentery. Extract of flowers, fruits and leaves are applied on skin burns. Stem extract reduces sugar level in blood.
*Ocimum tenuiflorum* L., Lamiaceae, AGS-24	47	Krishna Tulsi (N)/Kalo Tulsi (M)/Tilsi (Th)/Herb/Wild/Easily	Entire plants/Herb	Decoction of, plant is given in fever, cough, cold, headache, nausea, Diarrhoea, dysentery and skin diseases. Leaf juice is used as ear drops in earache. Leaf powder with honey is given orally in diabetics.
*Phragmites vallatoria* (L.) Veldkamp, Poaceae, AGS-64,	21	Narkat (N//M/Th)/Herb/Wild/Easily	Root/Herb	*Root decoction is given orally as refrigerant, diuretic and diaphoretic.
*Phyllanthus emblica* L., Euphorbiaceae, AGS-17	45	Amala (N)/Aura (Th)/Tree/cultivated,	Bark juice and fruit.	Bark juice is given orally in dysentery, constipation, and body ache. Fruits decoction is given orally in shore throat and as tonic.
*Polygonum barbatum* L., Polygonaceae, AGS-26,	13	Pire Jhar (N)/Bisnair (Th)/Herb/Wild/Easily	Entire plant	Poultice is applied externally on swelling parts of the body. *Root is given orally as astringent and cooling agent. Leaf decoction is applied externally to wash ulcers.
*Rauvolfia serpentina* Benth. ex Kurz Apocynaceae, AGS-37	47	Sarpagandha (N)/Chand maruwa (M)/Dhaldhaliya (Th)/Shrub/Rare in southern parts and Cultivated in northern parts	Leaf & root	Dried root powder is given orally to reduce high blood pressure. Root infusion is given orally in intestinal disorders. *Leaf juice is used as remedy for the removal of opacities of cornea. Root paste is applied on cuts, wounds, or boils.
*Rumex nepalensis* Spreng*,* Polygonaceae, AGS-61	24	Halhale Sag (N/Th)/Ban Palungo (M)/Herb/Wild/Easily	Entire plant	Seeds infusion is used in mouth disorders. Root paste applied externally on joint pains and wounds. Fresh leaf extract and sap is applied on cuts, wounds, and swellings.
*Ricinus communis* L., Euphorbiaceae, AGS-4	31	Ander (N)/Redi, Yamyam (Th)/Shrub/Wild/Easily	Root and seed	Root juice is given orally in diarrhoea, dysentery, and skin diseases. *Seed oil is applied as massage for babies and also applied on sole to relief from burning sensation. Seed oil is given orally in constipation and rheumatic pain.
*Shorea robusta* C.F. Gaertn., Dipterocarpaceae, AGS19	20	Sal (N)/Sakhuwa (Th)/Tree/Wild and cultivated	Root, Bark, resin and seed	Decoction is given orally in Diarrhoea and bloody dysentery. *Bark juice is used as eardrop in earache.
*Solanum nigrum* L., Solanaceae, AGS-2	36	Kali gedi (N)/Kamai (Th)/Herb/Wild/Easily	Entire plant	*Unripe fruits paste is applied on ringworm. Ripe fruits are given orally in constipation. Plant paste is applied externally in headaches and joint pain. Plant juice is given orally in liver enlargement, dysentery and fever
*Syzygium cumini* (L.) Skeels, Myrtaceae, AGS-29	40	Phader (N)/Jamuno (M)/Jam (Th)/Tree/Wild and cultivated	Bark and fruit	Bark juice is given orally in Diarrhoea, dysentery, cut and wounds. Fruits are given orally in indigestion and constipation. Bark, Leaf and seed powder is given orally to reduce sugar level in blood.
*Terminalia bellirica* (Gaertn.) Roxb., Combretaceae, AGS-39	36	Barro (N)/Baheda (Th)/Tree/cultivated	Stem bark and fruit.	Bark juice is applied externally in cut, wounds, and skin diseases. Fruits powder is given orally in cough, cold, respiratory problems, fever, and indigestion.
*Terminalia chebula* Retz., Combretaceae, AGS-64	36	Harro (N)/Harad (Th)/Tree/cultivated	Stem bark and fruit.	*Bark is chewed in urinary problems. Fruits are given orally in cough, cold, respiratory troubles, fever, and indigestion and stomach problems.
*Tribulus terrestris* L., Zygophyllaceae, AGS-47	16	Gokharu, Gaikhure Jhar (N)/Herb/Wild/Rare	Entire plant	*Decoction is given orally in urinogenital tract infection.
*Vitex negundo* L., Vitaceae, AGS-69	38	Simali (N)/Shrub/Wild/Easily	Leaf juice and bark	Leaf juice is given orally in cough, cold, sinusitis, fever, stomach problems, and rheumatic swellings. Bark paste is applied on boils.
*Zingiber officinale* Roscoe, Zingiberaceae	73	Aduwa (N)/Suntho (Th)/Herb/cultivated	Rhizome	Rhizome juice is given in cough, cold, fever, indigestion, and constipation. Rhizome is chewed in bronchial infections.
*Ziziphus mauritiana* Lam., Rhamnaceae, AGS-43	51	Bayer (N)/Tree/Wild/easily	Stem bark and fruit.	The juice of bark is given orally to treat Diarrhoea and dysentery. Ripe fruit are given orally in indigestion, constipation and other stomach problems.

^#^Exact doses and duration of treatment are considered as intellectual property of informants, so as per their request this information is not included in the present paper. F = Frequency of citation; N = Nepali; M = Magar; Th = Tharu. * = New reports.

**Table 3:** Different ailments of study area grouped under different ailment categories with their biomedical terms and factor of informants’ consensus

**Ailment categories**	**Biomedical terms**	^**1**^**N**_**TAXA**_	^**2**^**N**_**UR**_	^**3**^**F**_**IC**_
Gastro-intestinal disorders	Constipation, Diarrhoea, Dysentery, Nausea, Indigestion, Vomiting, Stomach-ache, Gastric trouble, Loss of appetite, Intestinal worms, colic pain, Flatulence, piles	41	836	0.95
Dermatological disorders and cosmetics	Cut, Wounds, Boils, Pimples, Skin rushes, Ringworm, Scabies, Leprosy, Skin burns, Skin blemishes, Ecto-parasites, Skin diseases, Hair problems, Body Inflammation	34	591	o.94
Respiratory diseases	Common cold, cough, asthma, Bronchitis, Chest pain, Lung disorders	13	235	0.94
Fevers	Ordinary fever, diaphoretic Malaria, Typhoid,	11	213	0.95
Ureno-genital problems	Sexual debility, Infertility, Leucorrhoea, Gonorrhoea, Menstrual disorders, Frequent urination, Diuretic, aphrodisiac	14	190	0.93
Ear, Nose, Throat problems	Earache, Throat shore, Nose bleeding, Sinusitis	12	205	0.94
Oral and dental disorders	Toothache, Mouth shore,	8	141	0.95
Mental disorders	Mental tonic, memory tonic, Epilepsy	4	44	0.93
Skelto-muscular pain and swelling	Body ache, muscular pain, Sprain, Strain, Rheumatism, Arthritis, Head ache, Joint pain, swelling	16	245	0.93
Cardio-vascular disorder	Cardiac, blood pressure	2	35	97
Other	Fracture, Tonic, Lactation, Easy delivery, Tumour, Diabetes, Cooling agent, stimulant and Eye problems	20	414	0.95
Total		175 ^4^	3149	0.94

^1^N_UR_ = number of use report in a particular illness category and

^2^N_TAXA_ = number of taxa used to treat that particular category by informants.

^3^F_IC_ **=** Factor of informants consensus = N_UR_ – N_TAXA_/(N_UR_-1), value of F_IC_ ranges from 0 to 1, high value shows agreement and low value shows disagreement among informants about the uses of taxa for the treatment of particular ailment category.

^4^ Many plants are used for more than one ailment category.

**Table 4:** Symptoms of the diseases given by the tribes in terai forest of western Nepal and their equivalent bio-medical terms

**Ailment categories**	**Bio-medical terms**	**Local terms**
Gastro-intestinal disorders	Constipation	Kabjiyat hunu/Pet safa na hune
	Diarrhoea	Pani jasto patlo dish hune
	Dysentery	Aau pareko
	Nausea	Kamjori hune wak-wak lagne
	Indigestion	Khana apach hune
	Vomiting	Banta/Ulti hune
	Stomachache	Pet dukhne
	Gastric trouble	Pet dhadiyeko
	Loss of appetite	Khana ruchi na lagne/Bhok na lagne
	Intestinal worms	Pet ma juka parnu
	Colic pain	Tallo pet dukhne
	Flatulence	Bayu gola le pet dukhne
	Piles	Disha garne thaun ma mashu palaune
Dermatological disorders & cosmetics	Cuts	Katiyeko
	Wounds	Ghau
	Boils	Pilo, Khatira
	Pimples	Dandiphore
	Skin rushes	Chhala ma chilaune, rato dana hune
	Ringworm	Daad hune, Chhala ko rog
	Scabies	Luto, Kanaune rog, Khujali hune
	Leprosy	Kushta rog
	Skin burns	Ghamle chhala dadeko
	Skin blemishes	Chhala ma hune rog
	Ecto-parasites	Jumra parnu, juka lagnu
	Skin diseases	Charma rog
	Hair problems	Rauko rog, Kapal ko samsya
	Body inflammation	Sarir sunnine ra polne
Respiratory diseases & Fever	Common cold	Chiso lageko
	Cough	Khoki lageko
	Asthma	Dam rog bhayeko, Swash phulne rog
	Bronchitis	Ghanti ko rog
	Chest pain	Chhati Dukheho
	Lung disorders	Fokswo ko rog
	Ordinary fever	Samanaya rog
	Diaphoretic	Pasina bagaune rog
	Malaria	Aulo Jwaro
	Typhoid	Miyadi Jwaro
Ureno-genital disorders	Sexual debility	Saririk Kamjori
	Infertility	Youn Durbalta
	Leucorrhoea	Swet Pradar/Yoni bat seto pani bagne
	Gonorrhoea	Yoni bat ganaune pani jasto aaune/Estree haru ma youn rog
	Menstrual disorders	Nachune huda ko rog/Mahinwari huda lagne rog
	Frequent urination	Pishab aayee rakhne
	Diuretic	Pishab kholne
	Aphrodisiac	Youn bardhak/Youn ko tagat
Ear, Nose, Throat problems	Earache	Kan dukhne
	Throat sore	Ghanti baseko
	Nose bleeding	Nak bat ragat bagne
	Sinusitis	Pinas bhayeko
Oral & Dental disorders	Toothache	Dant Dukheko
	Mouth sore	Mukhma ghau, dana hune
Mental disorders	Mental tonic	Buddhi badhaune aushadhi
	Memory tonic	Smaranshakti badhaune aushadhi
	Epilepsy	Chhare rog/Mirgi rog
Skeleto-muscular pain & swelling	Body-ache	Jiu dukheko
	Muscular pain	Manspeshi haru dukheko
	Sprain	Markeko
	Strain	Tanaw bhayeko
	Rheumatism	Baath bhayeko
	Arthritis	Jorni dukheko
	Headache	Tauko dukheko
	Joint pain	Jorniharu dukheko
	Swelling	Sunniyeko
Cardiovascular disorders	Cardiac tonic	Mutu lai tagat dine aushadhi
	Blood pressure	Rakta chaap bhayeko
Others	Fracture	Haadi bhachiyeko
	Tonic	Tagat dine aushadhi
	Lactation	Dudh badhaune
	Easy delivery	Sajilai sutkeri garaune
	Tumour	Mashu badheko
	Diabetes	Chinirog/Madhumeh bhayeko
	Cooling agent	Shitalta dine aushadhi
	Stimulant	Uttejana badhaune aushadhi
	Eye problems	Aankh ko rog

### Growth forms, plant parts used, method of collection, processing and administration

**Figure 2:**
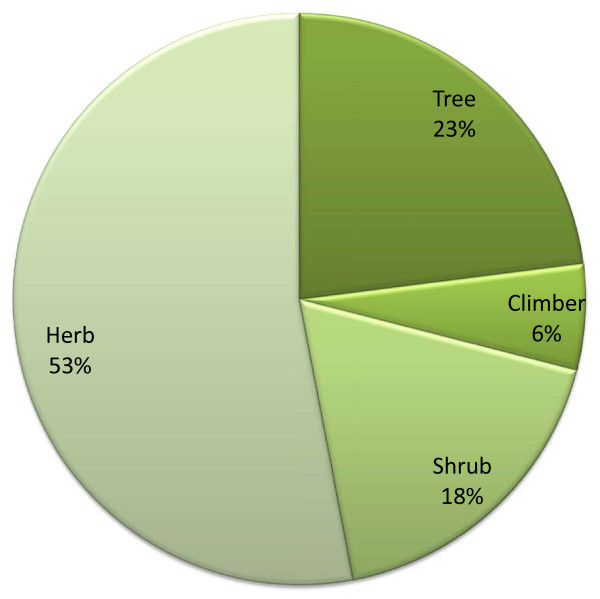
Life form of plants used as medicinal plants in Terai forest of western Nepal.

**Figure 3:**
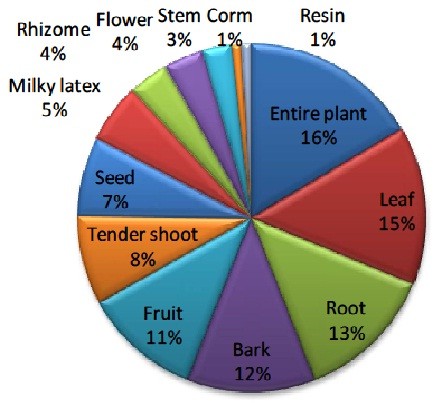
Plant parts used for the management of various healthcare problems in Terai forest of western Nepal.

**Figure 4:**
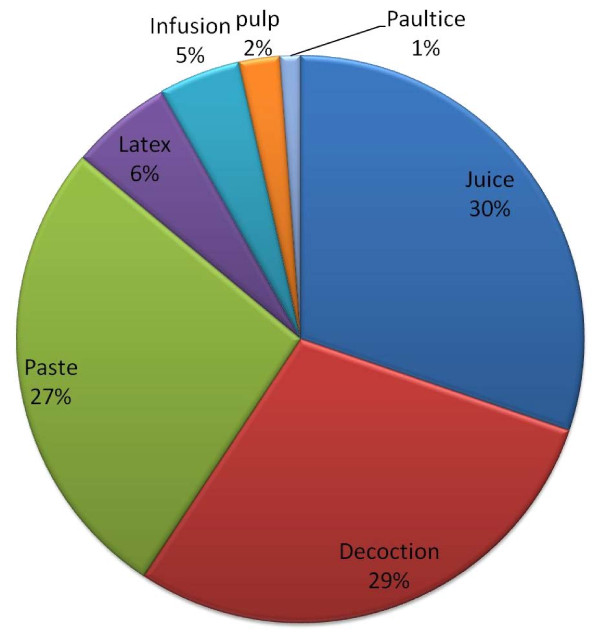
Processing of medicinal plant/part(s) for crude drug preparation in the study area.

Out of 66 medicinal plants recorded from study area, highest number of plants belongs to herb (53%) followed by tree, shrubs and climber (Figure 2). Higher uses of herbs for medicinal purposes may be due to easy availability and high effectiveness in the treatment of ailments in comparison to other growth forms. Almost every plant parts are used for the medication either singly or in combination with other plants. Entire plant is used in the majority of cases followed by leaf, root and bark (Figure 3). Plant parts used as medicine is collected by healer themselves from natural resources. Generally fresh parts are collected for use from nature. Various plant parts are collected in different seasons at different stage of maturity and are dried in shade and stored in dry places away from direct sunlight for their use during off season/unavailability. As far as mode of use and administrations are concerned majority of the plants are used in form of juice, followed by decoction (Figure 4). Majority of the medicinal formulations are administrated orally in ailment categories other than dermatological. In dermatological problems plants are administrated topically as well as orally.

### Identification of new claims and reliability of reported claims

Reported uses of various medicinal plants were compared with previously published ethnobotanical literatures in Nepal and adjoining areas of India [2-20,25,30] which identifies new medicinal uses of *Acacia catechuAcalypha indica, Achyranthes aspera, Aegle marmelos, Aloe vera, Artemisia indica, Bauhinia variegata, Bombax ceiba, Calotropis gigantea, Carica papaya, Citrus limon, Colocasia esculentaCoriandrum sativum, Curcuma amadaCuscuta reflexa, Cynodon dactylon,Dalbergia sissoo, Datura metel,Dendrocalamus hamiltonii, Dioscorea pentaphylla, Ficus benghalensis, Gloriosa superbaIpomoea aquatica, Ipomoea batatas, Ipomoea carnea Jacq. ssp. fistulosaLagenaria siceraria, Lepidium sativum, Linum usitatissimum, Malva parvifloraMentha spicata, Mimosa pudica, Mucuna pruriens,Phragmites vallatoriaPolygonum barbatumRauvolfia serpentina, Ricinus communis Shorea robusta, Solanum nigrum, Terminalia chebula,* and *Tribulus terrestris* are reported for the first time in Nepal and adjoining areas of India. Some of the medicinal plants reported during the present study were reported for biological activities and bioactive constituents responsible for their therapeutic properties [7,17,46-50] which justify and validate the usages of these species for medicinal purposes in the study area.

### Consensus of agreement about uses of medicinal plants among informants

To gain credibility, scientific studies that utilize traditional knowledge must be reliable. In ethnobotanical studies, consensus analysis provides a measure of reliability for any given claim providing reliable evidence. The product of F_IC_ ranges from 0 to 1. High value of F_IC_ indicates the agreement of selection of taxa between informants, whereas a low value indicates disagreement [51]. Recently consensus analysis has been used as an important tool for the analysis of ethnobotanical data [19,22,51-58]. In the study area the informants’ consensus about usages of medicinal plants ranges from 0.93 to 0.97 with an average value of 0.94 (Table 3), which shows high level of agreements among the informants. The high level of consensus among the informants about the usages of medicinal plants for the treatment and prevention of various diseases and ailments prevalent in the study area suggests that the ethnomedicinal uses of plants are currently in practice in the study area.

### Availability of medicinal plants in terai forest, conservation efforts and needs

As for as availability of medicinal plants is concerned 39% medicinal plants are cultivated for food, fruit, spices and trade; thus are easily available for medicinal purposes. Majority of the 61% wild medicinal plant species are available without difficulty in the study area except *Acacia catechu, Bacopa monnieri, Bombax ceiba, Drymaria diandra, Rauvolfia serpentina and Tribulus terrestris* which are available with difficulty and needs to be conserved for future use. Unfortunately, neither local inhabitants nor Government is making serious efforts for conservation of medicinal plants in the study area. Unsustainable collection of generative and vegetative parts of medicinal plants from natural resources reduces their population as well as decrease multiplication and regenerative power. There is an urgent need to create awareness among the inhabitants of the study area about sustainable collection, conservation, domestication, small scale (home garden for personal use) as well as large scale (for trade) cultivation of medicinal plants. This will also improve the socio-economic condition of the inhabitants as well as reduce pressure on natural resources.

### Knowledge about traditional healing system and its transfer from one generation to other

Bhagirathi Tharu, Mandali Tharu and Khadanand Poudyal are the main expert from the study area. These experts are working in this field since more than 30 years. Though there is a sub health post with less equipped facility in Shankar Nagar VDC and the modern hospital facilities are available in Butwal municipality which is near about 10 km far from Shankar Nagar VDC. The tribal people of the study area prefer traditional medicinal practice to the modern medicinal system because they know more about the medicinal plants which are easily available in their local area and herbal formulations are cooperatively cheaper and free from side effects. The tribal communities of the study area are not exception to the present stream of modernization and the traditional medicinal practice seems to be disappearing among the tribal communities of the study area. During present study it was found that the knowledge about utilization of medicinal plant species is generally accumulated by observation and experiences and transferred to the next generation by words of mouth. Our finding was similar to findings in other parts of India and abroad [24-30]. As indigenous knowledge on usages of medicinal plants is transmitted without any systematic process, and younger generations of the tribes are not interested in traditional healing system because it has no/very little scope for money, so they engage themselves in other occupations. Thus, it is certain that such knowledge is at the risk of disappearance in the future [21].

## Conclusion

Present study revealed that the local traditional healers of Rupandehi district, western Nepal are rich in ethnomedicinal knowledge and majority of people rely on plant based remedies for common health problems like headache, body ache, constipation, indigestion, cold, fever, diarrhea, dysentery, boils, wounds, skin diseases, urinary troubles, fractures, round worms, etc. The survey also revealed that all the traditional healers have strong faith on ethnomedicines although they were less conscious about the documentation and preservation of ethno medicinal folklore and medicinal plants. The group discussion and personal interviews show that youngsters of both Tharu and migrant society are less aware about the use of ethnomedicines; our findings are similar to reports from India [58]. On the other hand, traditional healers who are the main repository of ethno medicinal knowledge claim extreme secrecy over their ethnomedicinal knowledge. The traditional healers have strong believe that if they disclose the secrecy about the medicinal properties of particular plant all the medicinal potentialities of the plant will be lost and the remedy will not work properly.

## Competing interest

The authors declare that they have no competing interests.

## Authors’ contributions

AGS, AK and DDT developed and designed the research study. AGS conducted field survey work, collected data and prepared draft of the manuscript. AK conducted statistical analysis and revised the manuscript. All authors have read and approved the final manuscript.
